# Resveratrol Rescue Indoxyl Sulfate-Induced Deterioration of Osteoblastogenesis via the Aryl Hydrocarbon Receptor /MAPK Pathway

**DOI:** 10.3390/ijms21207483

**Published:** 2020-10-11

**Authors:** Wen-Chih Liu, Jia-Fwu Shyu, Yuh-Feng Lin, Hui-Wen Chiu, Paik Seong Lim, Chien-Lin Lu, Cai-Mei Zheng, Yi-Chou Hou, Po-Han Chen, Kuo-Cheng Lu

**Affiliations:** 1 Graduate Institute of Clinical Medicine, College of Medicine, Taipei Medical University, Taipei 110, Taiwan; wayneliu55@gmail.com (W.-C.L.); linyf@shh.org.tw (Y.-F.L.); leu3@tmu.edu.tw (H.-W.C.); 11044@s.tmu.edu.tw (C.-M.Z.); athletics910@gmail.com (Y.-C.H.); 2 Division of Nephrology, Department of Internal Medicine, Taipei Hospital, Ministry of Health and Welfare, New Taipei City 242, Taiwan; 3 Department of Biology and Anatomy, National Defense Medical Center, Taipei 114, Taiwan; shyujeff@mail.ndmctsgh.edu.tw (J.-F.S.); Rabelais1017@hotmail.com (P.-H.C.); 4 Division of Nephrology, Department of Internal Medicine, Shuang Ho Hospital, Taipei Medical University, New Taipei City 235, Taiwan; 5 TMU Research Center of Urology and Kidney, Taipei Medical University, Taipei 110, Taiwan; 6 Division of Nephrology, Department of Internal Medicine, Tungs’ Taichung MetroHarbor Hospital, Taichung City 435, Taiwan; jamespslim@gmail.com; 7 Division of Nephrology, Department of Medicine, Fu Jen Catholic University Hospital, School of Medicine, Fu Jen Catholic University, New Taipei City 242, Taiwan; janlin0123@gmail.com; 8 Division of Nephrology, Department of Internal Medicine, School of Medicine, College of Medicine, Taipei Medical University, Taipei 110, Taiwan; 9 Division of Nephrology, Department of Medicine, Cardinal Tien Hospital, School of Medicine, Fu Jen Catholic University, New Taipei City 231, Taiwan; 10 Division of Nephrology, Department of Medicine, Taipei Tzu Chi Hospital, Buddhist Tzu Chi Medical Foundation, New Taipei City 231, Taiwan

**Keywords:** aryl hydrocarbon receptor, indoxyl sulfate, osteoblast, chronic kidney disease, Runx2, mitogen-activated protein kinase

## Abstract

Indoxyl sulfate (IS), a uremic toxin derived from dietary tryptophan metabolism by the gut microbiota, is an endogenous aryl hydrocarbon receptor (AhR) agonist and a key player in bone remodeling. Resveratrol (RSV), an AhR antagonist, plays a protective role in shielding against AhR ligands. Our study explored the impact of IS on osteoblast differentiation and examined the possible mechanism of IS in controlling the expression of osteoblastogenesis markers through an in-depth investigation of AhR signaling. In vivo, we found histological architectural disruption of the femoral bones in 5/6 nephrectomies of young adult IS exposed mice, including reduced Runx2 antigen expression. RSV improved the diaphysis architecture, Runx2 expression, and trabecular quality. In vitro data suggest that IS at 500 and 1000 μM disturbed osteoblastogenesis through suppression of the ERK and p38 mitogen-activated protein kinase (MAPK) pathways, which were found to be downstream of AhR. RSV proved to ameliorate the anti-osteoblastogenic effects of IS through the inhibition of AhR and downstream signaling. Taken together, we demonstrated that the IS/AhR/MAPK signaling pathway plays a crucial role in the inhibition of osteoblastogenesis, and RSV has a potential therapeutic role in reversing the IS-induced decline in osteoblast development and suppressing abnormal bone turnover in chronic kidney disease patients.

## 1. Introduction

Indoxyl sulfate (IS), an organic anion uremic toxin, is a protein-bound retention solute [1]. The accumulation of IS leads to harmful consequences, including immune dysfunctions and cardiovascular diseases [2,3]. IS works as a bone toxin by decreasing osteoclast differentiation [4,5] and promoting osteoblast apoptosis [6]. Therefore, it is important to consider bone disorders in chronic kidney disease (CKD) patients as being attributed to both reduced bone quality and quantity by IS. Nii-Kono et al. suggested that higher IS levels may play a role in low bone turnover and adynamic bone disease because they cause skeletal resistance to Parathyroid Hormone (PTH) in CKD patients [7].

Resveratrol (RSV) is a polyphenolic compound that is abundant in natural plants, including grapes, berries, peanuts, and dark-skinned fruits [8]. RSV has the benefit of promoting the intestinal epithelial tight junction [9], modulating gut probiotic microbiota [10,11], and reducing the synthesis of IS by inhibiting hepatic sulfotransferase in the liver [12] to provide cardiovascular protective functions. Considerable evidence demonstrates that the biological properties of RSV are related to the mitogen-activated protein kinase (MAPK) pathway, an ancient evolutionarily conserved pathway that exists in all eukaryotes and regulates cell proliferation, differentiation, and survival. Recent research has demonstrated that the MAPK pathways are involved in human bone development [13]. The therapeutic effect of RSV on bone formation via MAPK signaling; however, is not fully understood [14].

Runt-related transcription factor 2 (Runx2) is an important transcription factor for osteoblast differentiation, and its fundamental role is in regulating bone formation [15]. The activation of Runx2 is controlled by interactions with other additional nuclear factors and conducting posttranslational modifications such as phosphorylation. Recent studies have shown that the ERK1/2 and p38 signal pathways promote osteoblast differentiation, inducing the phosphorylation and activation of Runx2 [16,17,18].

The aryl hydrocarbon receptor (AhR) is a member of the Per-Arnt-Sim superfamily. The inactive form of AhR combines with the 90 kDa heat shock protein (HSP90), AHR-interacting protein (AIP), co-chaperone p23, and protein kinase SRC in the cytosol [19]. When AHR binds to one of its ligands, such as 2,3,7,8-tetrachlorodibenzo-p-dioxin (TCDD) (exogenous ligand) [20] or IS (endogenous ligand) [20,21], it undergoes a conformational change to reveal the nuclear transcription locus and then heterodimerizes with the aromatic hydrocarbon receptor nuclear transfer protein (ARNT) in the nucleus [22] to regulate the expression of target genes—e.g., cytochrome P450 family 1 subfamily A member 1 (CYP1A1) and CYP1B1. Korkalainen et al. found that osteoblast differentiation can be inhibited by AhR ligands [23]. In our previous article, we demonstrated that IS can inhibit osteoclastogenesis through the AhR/NFATc1 signaling pathway [4].

Recent studies have suggested that RSV, as an Aryl hydrocarbon receptor (AhR) antagonist [20], may play a protective role in shielding against AhR ligands and blocking the production of CYP1A1 and CYP1B1 [24]. However, RSV’s effects in the rescue of IS-induced osteoblast dysfunction in chronic kidney disease (CKD) have not been well-characterized. Therefore, we inspected the in vivo effects of an IS-induced pathological bony architecture scenario and studied the molecular mechanism of osteoblast differentiation and maturation through the AhR/MAPK/Runx2 signaling pathway in vitro to explore the possible RSV benefits on bone quality and constitution under exposure to IS.

## 2. Results

### 2.1. RSV Attenuates the Effect of Renal Function Insufficiency on Bone Formation In Vivo

**Figure 1:**
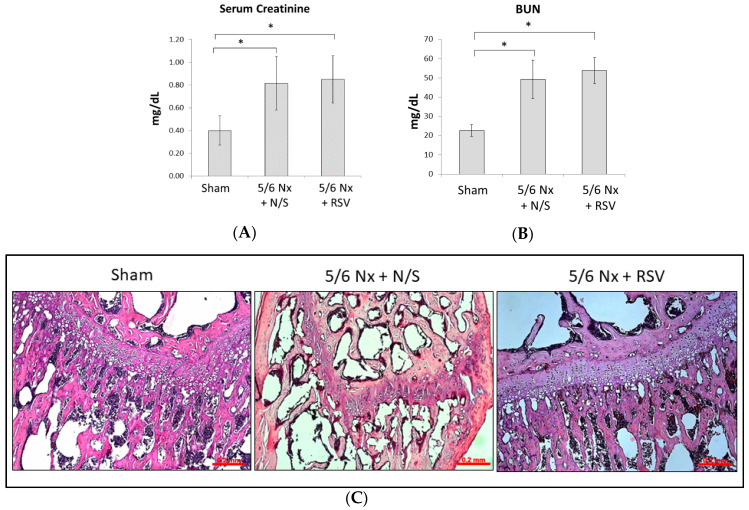

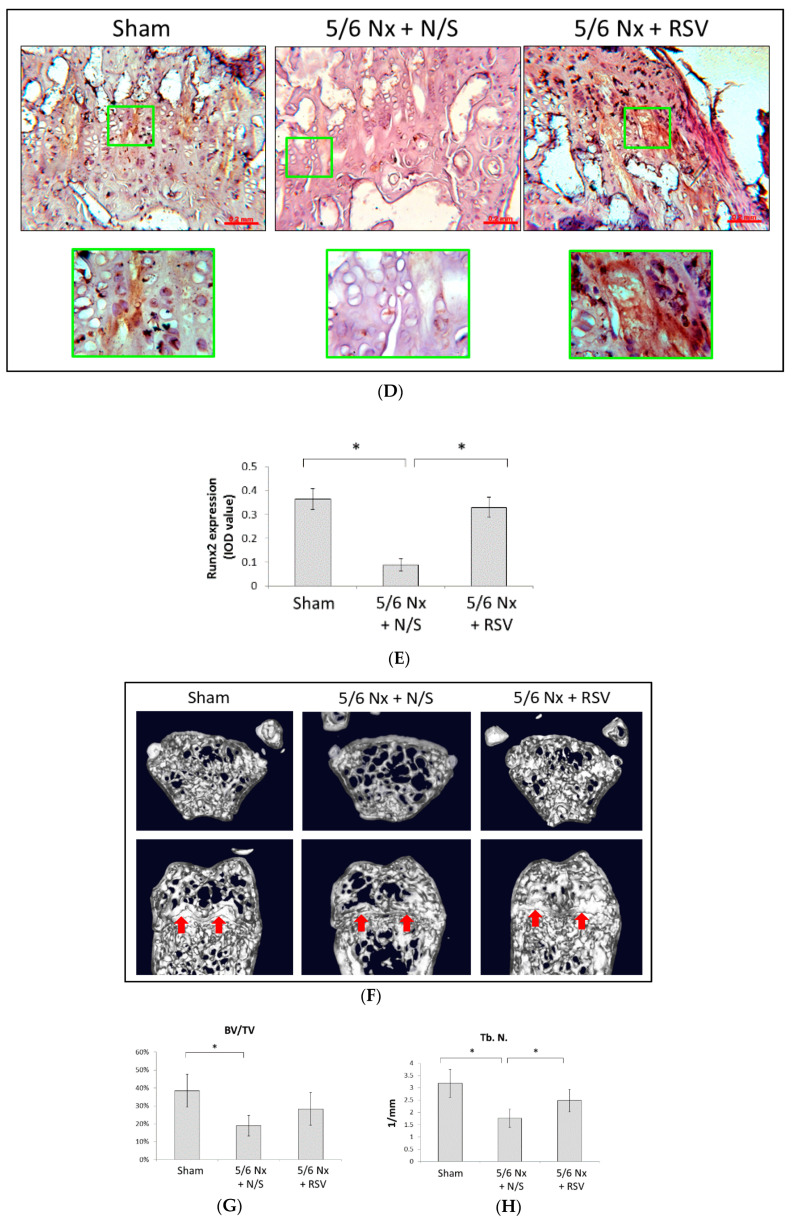

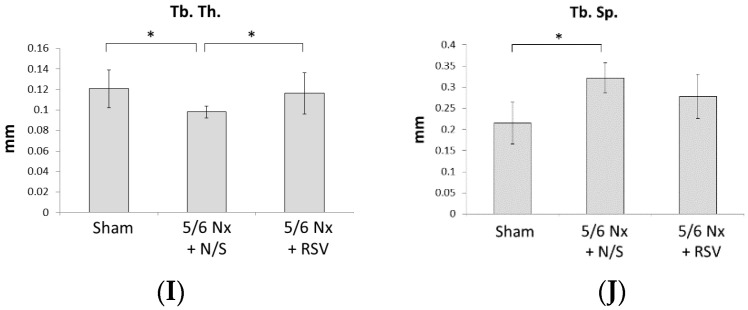
Resveratrol (RSV) ameliorates the impaired bone quality of mice with impaired renal function. BALB/c male mice were divided into three groups: sham + normal saline (N/S) treatment; 5/6 Nephrectomy (Nx) + N/S treatment; and 5/6 Nx + RSV treatment (*n* = 6 in each group) (**A**,**B**). After 4 weeks of surgery and before injection treatment, the serum levels of creatinine and blood urea nitrogen (BUN) of the 5/6 nephrectomy groups were significantly elevated compared to the sham group. (**C**) After 4 weeks of injections, H&E staining applied to the bone tissue showed the chondrocyte rows of the 5/6 Nx + RSV group to be more orderly than those of the 5/6 Nx + N/S group. Scale bar = 0.2 mm. (**D**) Immunohistochemical (IHC) staining with DAB represents the preservation of Runx2 expression (brown particles). (**E**) The relative interest of density (IOD) values of Runx2 in bone tissue showed that the 5/6 Nx + N/S group has the lowest expression. Scale bar = 0.2 mm. (**F**) The micro-CT images showed that the cancellous bone tissue and epiphyseal plate (red arrows) in the distal femur metaphysis of 5/6 Nx + RSV group were increased and thicker, respectively, compared to the 5/6 Nx + N/S group. (**G**) The micro-CT analysis data reveal that the bone volume over total volume (BV/TV) in the 5/6 Nx + N/S group was significantly decreased in comparison with the sham group, but the 5/6 Nx + RSV group shows an increasing trend compared to the 5/6 Nx + N/S group. (**H**,**I**) In terms of trabecular number (Tb. N) and trabecular thickness (Tb.Th), the values of the 5/6 Nx + N/S group significantly decreased compared to the sham group, and those of the 5/6 Nx + RSV group significantly increase compared to the 5/6 Nx + N/S group. (**J**) In terms of trabecular separation (Tb.Sp), the values for the 5/6 Nx + N/S group here are significantly increased compared to the sham group, but the 5/6 Nx + RSV group showed a decreasing trend compared to the 5/6 Nx + N/S group. Data are represented as the mean ± SEM (*n* = 6). * Indicates differences that are considered significant between the indicated groups (*p* < 0.05).

To evaluate the injured renal effects of mice undergoing 5/6 nephrectomy (Nx) surgery, the serum levels of creatinine and blood urea nitrogen (BUN) were measured 4 weeks after surgery. The levels of serum creatinine in the 5/6 Nx with RSV treatment group (0.82 ± 0.23 mg/dL, *n* = 6) and normal saline (N/S) treatment group (0.85 ± 0.21 mg/dL, *n* = 6) significantly increased compared to the sham group (0.40 ± 0.13 mg/dL, *n* = 6; *p* < 0.05, respectively), as did the levels of BUN (49.32 ± 9.94 mg/dL or 53.78 ± 6.78 mg/dL vs. 22.63 ± 3.08 mg/dL; *p* < 0.05, respectively) (Figure 1A,B). This result shows that the 5/6 Nx procedure successfully induced impaired renal function in mice.

After treatment of mice with impaired renal function using N/S or RSV for 4 weeks, we evaluated the bone histology with hematoxylin and eosin staining (H&E staining), as well as immunohistochemical staining. Under a microscope, different zones of ossification can be readily seen in the normal mouse epiphyses (Figure 1C, left). H&E staining of the proximal femoral epiphysis showed characteristics of histological architectural disruption in the 5/6 Nx + N/S group, including inadequate chondrocyte hypertrophy, an irregular array in the chondrocyte, and an unclear calcified cartilage matrix surrounding the dying chondrocytes near epiphyseal plate (Figure 1C, middle). RSV administration preserved the epiphyseal architecture and reduced the irregular alignment of the chondrocytes (Figure 1C, right). The bone tissue expression of Runx2, an important marker of osteoblasts, was evaluated by Immunohistochemical (IHC) 3,3’-Diaminobenzidine (DAB) staining. The preservation of Runx2 in the Sham group and 5/6 Nx + RSV group was more pronounced than that in the 5/6 Nx + N/S group (Figure 1D,E). Based on the bone morphology and histological antigenicity pictures, the performance of osteoblasts is reduced in 5/6 Nx + N/S and enhanced in 5/6 Nx + RSV.

The radiation (micro-CT) images demonstrated that the 5/6 Nx + N/S group had the thinnest epiphyseal plate and the highest cancellous bone mass porosity (Figure 1F). However, mice with 5/6 Nx + RSV had a healthier epiphyseal plate and cancellous bone mass compared to mice with 5/6 Nx +N/S, which further supports the role of RSV in improving bone quality. The bone volume over total volume (BV/TV), trabecular number (Tb. N.), and trabecular thickness (Tb.Th.) of 5/6 Nx + N/S were decreased compared to the sham group (Figure 1G–I). By contrast, trabecular separation (Tb.Sp.) was significantly increased compared to the sham group (Figure 1J). However, RSV treatment for 4 weeks ameliorated the effects on Tb. N. and Tb.Th. in mice undergoing 5/6 Nx surgery (Figure 1H,I).

### 2.2. RSV Ameliorates Impaired Osteoblast Differentiation and Mineralization Induced by IS

**Figure 2:**
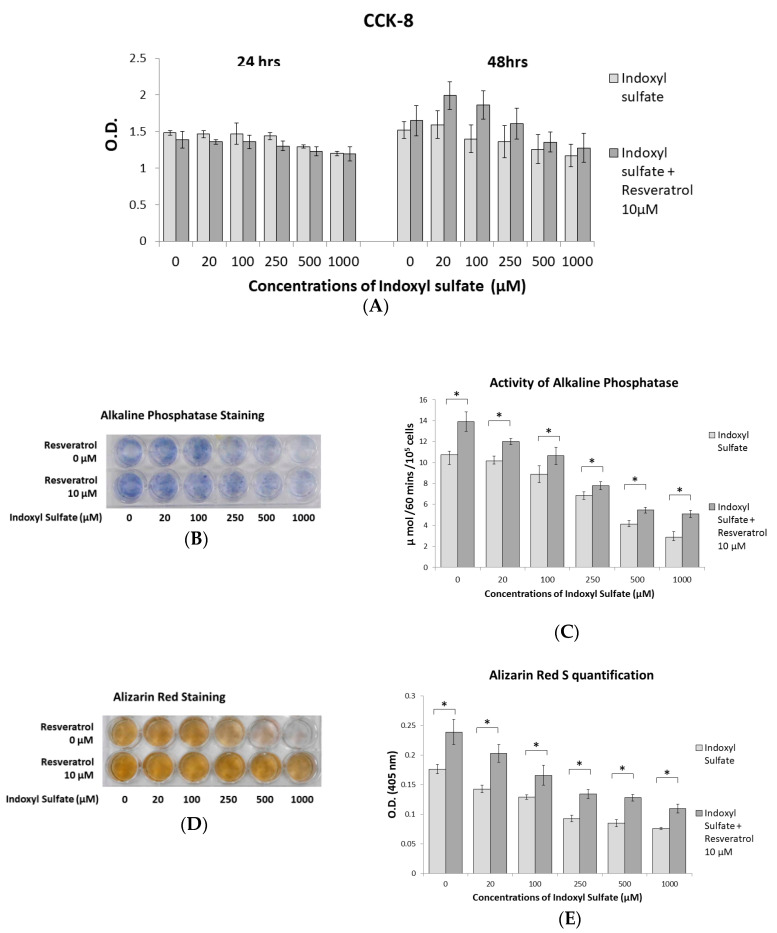
Effects of RSV on indoxyl sulfate (IS)-induced impaired osteoblast development. Primary osteoblast cells were cultured in an osteogenesis medium with IS at 0, 20, 100, 250, 500, and 1000 μM with or without 10 μM RSV. (**A**) A Cell Counting Kit-8 (CCK-8) assay was used to determine osteoblast cell viability. Cell viability is not significantly affected by different concentrations of IS or RSV at 24 and 48 h. (**B**) Primary osteoblast cells were cultured for 14 days, and alkaline phosphatase staining exhibited a dose-dependent decrease in response to increasing IS concentrations. The RSV treatment group increased the overall activity of alkaline phosphatase, as indicated by staining. (**C**) The alkaline phosphatase activity assay demonstrates that culturing the primary osteoblast cell culture for 14 days with RSV can significantly enhance alkaline phosphatase activity. (**D**) Primary osteoblast cells were cultured for 21 days; the level of Alizarin Red staining shows a dose-dependent decrease in response to increasing IS concentrations. The RSV-treated group improved the staining of Alizarin Red. (**E**) Alizarin Red staining quantification demonstrates that RSV treatment can significantly increase staining. Data are represented as the mean ± SEM (*n* = 6). * Indicates differences that were considered significant between the indicated groups (*p* < 0.05).

Primary osteoblast cells from neonatal mouse calvarial bone were cultured in a 96-well plate stimulated with IS at 0, 20, 100, 250, 500, and 1000 μM with or without 10 μM RSV for cell viability measurements at 24 and 48 h. Results of the CCK-8 assay, as shown in Figure 2A, demonstrated no significant difference in cell viability between IS doses and the groups with or without RSV. However, at 48 h, the group with RSV showed a trend of increasing cell viability. Alkaline phosphatase is a major early marker during the osteogenic differentiation of bone marrow-derived mesenchymal stem cells [25,26]. Alizarin Red staining is used to examine the mineralization of bone tissue [27]. Fourteen days of primary osteoblast cells were stained with alkaline phosphatase and the alkaline phosphatase activity was checked, while 21 days of cells were quantified according to Alizarin Red staining (Figure 2B–E). These results indicate that treatment with 10 μM RSV significantly alleviated the deterioration of osteoblasts caused by IS.

### 2.3. Prohibition of Osteogenesis Development through AhR Activated by IS Exposure

**Figure 3:**
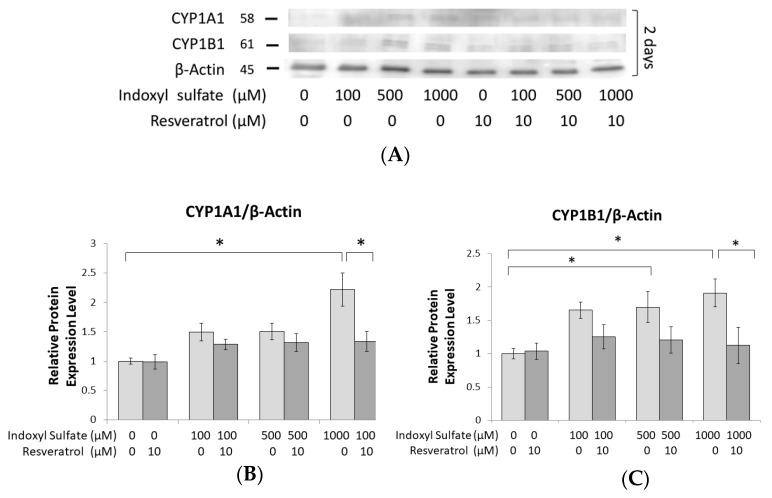

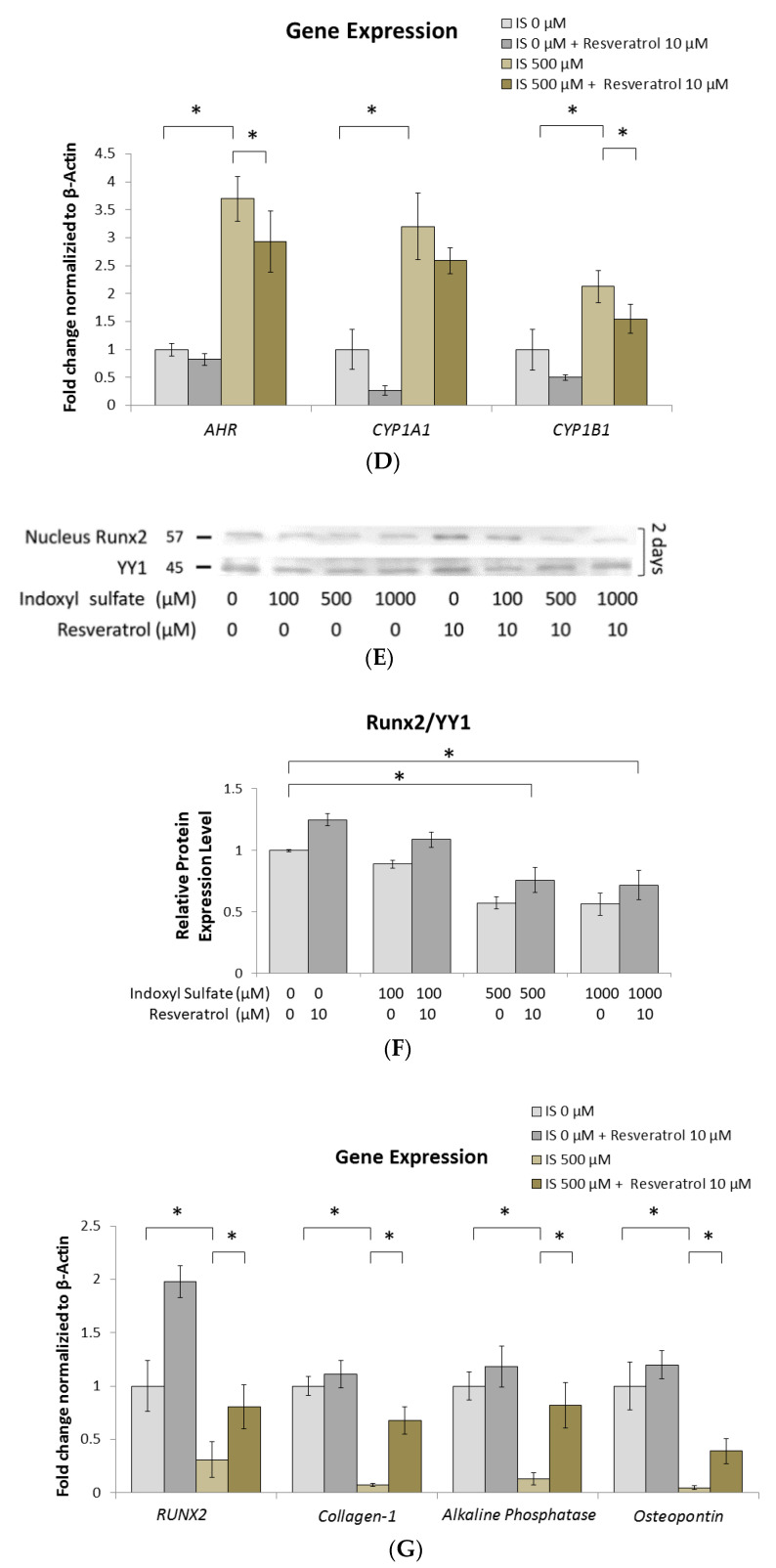
The effect of RSV on IS-induced actuate aryl hydrocarbon receptor (AhR) signaling and the rescue of osteoblast differentiation. MC3T3 E1 cells were cultured in an osteogenesis medium with IS at 0, 100, 500, and 1000 μM both with and without RSV for 2 days. (**A**–**C**) In whole cells, the protein expression of CYP1B1 significantly increased in response to 500 μM IS compared to the control group. At 1000 μM IS, the protein expression of both CYP1A1 and CYP1B1 significantly increased compared to the control group. Moreover, RSV significantly suppressed CYP1A1 and CYP1B1 expression. (**D**) At 500 μM IS, the expression of *AHR, CYP1A1,* and *CYP1B1* genes was significantly increased compared to their levels in the control group. In addition, when adding 10 μM RSV to the cells already treated with 500 μM IS, the expression of AhR and CYP1B1 was significantly suppressed. (**E**,**F**) In the nucleus, the protein expression of Runx2 was significantly decreased at 500 and 1000 μM IS. (**G**) At 500 μM IS, the expression of *RUNX2, collagen 1, alkaline phosphatase,* and *osteopontin* genes were all significantly lower compared to their expression in the control group. Data are represented as the mean ± SEM (*n* = 3). * Indicates differences that were considered significant between the indicated groups (*p* < 0.05).

To evaluate how IS affects AhR production, the expression of both the AhR target protein and mRNA in the MC3T3-E1 cell cultures was assayed 2 days after IS treatment. CYP1A1 and CYP1B1 have been identified as target genes for AhR transcriptional activity [28] and can precisely detect the activation status of AhR [29,30,31]. As seen in our results, the protein expression of CYP1A1 and CYP1B1 significantly increased under 500 and 1000 μM IS exposure (Figure 3A–C). Similar results were also observed for mRNA expression, with 500 μM IS significantly enhancing the expression of *AHR, CYP1A1,* and *CYP1B1* (Figure 3D). However, we found that treatment with 10 μM RSV appeared to terminate the elevated expression of AhR induced by IS (Figure 3A–D).

Furthermore, to better understand how the enhanced expression of AhR influences osteoblast development, we examined Runx2, which is a marker of pre-osteoblastic cells [32,33] and is able to enhance the expression of genes at early stages of osteoblast differentiation [34]. Runx2 protein expression was significantly suppressed in MC3T3-E1 cells with 500 and 1000 μM IS exposure in a dose-dependent manner on day 2 (Figure 3E). For mRNA, 500 μM IS significantly reduced the levels of *RUNX2* mRNA, while RSV significantly alleviated this suppression of IS (Figure 3F). In addition, the mRNA levels of other osteoblast developmental markers, such as *alkaline phosphatase*, *collagen 1*, and *osteopontin*, displayed similar responses to IS and RSV stimulation (Figure 3F). Collagen 1 represents the middle stage of osteoblast development markers as it is secreted by osteoblasts for the maturation of the extracellular matrix [35], and osteopontin represents a late stage marker, as it is actuated during bone remodeling and associated with embryonic bone formation [35,36,37]. These results show that the attenuation of osteoblast differentiation under IS exposure is dependent on AhR signaling.

### 2.4. IS Inhibits the Phosphorylation of ERK1/2 and p38 MAPK

**Figure 4:**
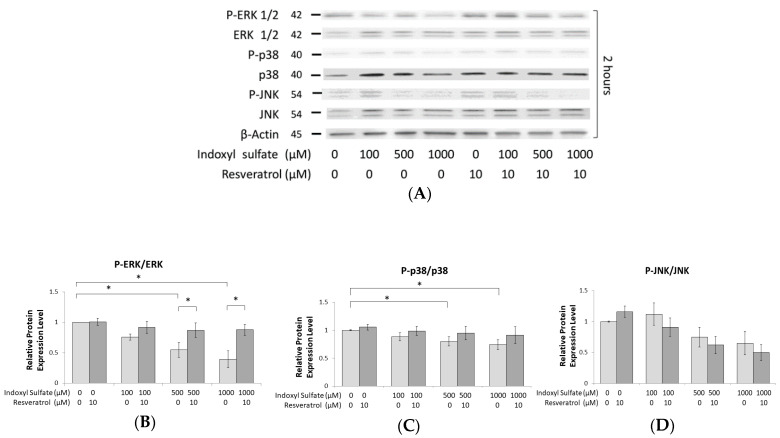

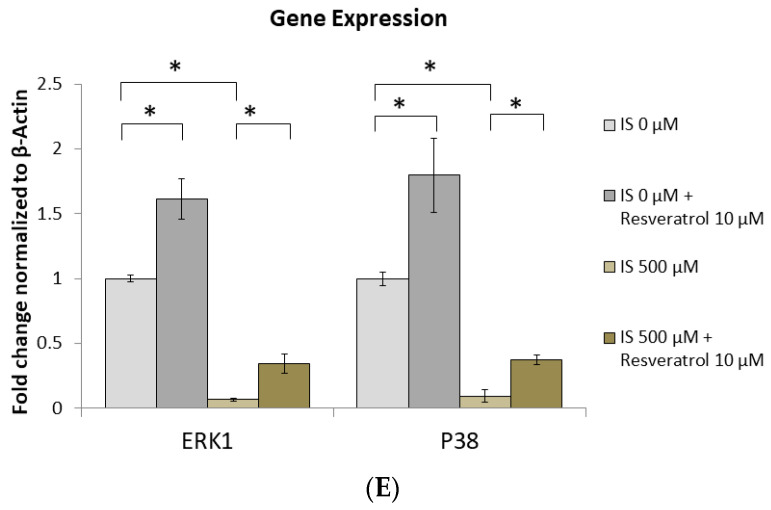
IS inhibits the phosphorylation of ERK and p38 MAPK. MC3T3 E1 cells were cultured in an osteogenesis medium with IS at 0, 100, 500, and 1000 μM with or without RSV for 2 h. (**A**–**D**) In whole cells, the phosphorylation of ERK1/2 and p38 decreased in a dose-dependent manner with the concentration of IS. At 500 and 1000 μM IS, ERK1/2 and p38 MAPK phosphorylation was significantly suppressed in comparison to the control group. RSV significantly alleviated ERK1/2 phosphorylation at 500 and 1000 μM IS. (**E**) At 500 μM IS, the expression of ERK1 and p38 genes was significantly reduced compared to their levels in the control group. When RSV is also added, the expression of ERK1 and p38 genes is significantly increased in response to IS treatment at 0 and 500 μM. Data are represented as the mean ± SEM (*n* = 3). * Indicates differences that were considered significant between the indicated groups (*p* < 0.05).

The MAPK pathway is an important mechanism of cell development; recently, it has been recognized that MAPKs regulate osteoblast differentiation, which establishes MAPKs as key signal transducers in the regulation of osteoblast development [38,39,40]. Therefore, we examined the effect of AhR signaling on the intracellular MAPK pathway under IS exposure. After MC3T3-E1 cells underwent 2 h of IS exposure, we found that there was no significant reduction in the phosphorylation of ERK1/2 and p38 MAPK when the IS dose was less than 100 μM. However, ERK1/2 and p38 MAPK phosphorylation was significantly reduced in a dose-dependent manner when the IS dose exceeded 500 μM. JNK did not exhibit the same trend, as its phosphorylation did not appear to be affected in response to IS (Figure 4A–D). Under the same doses of IS exposure, the expression of AhR was elevated; in contrast, the levels of phosphorylated ERK1/2 and p38/MAPK were suppressed (Figure 3A–C). In addition, RSV improved the suppressed phosphorylation of ERK1/2 and p38 MAPK after IS exposure (Figure 4A–D). From the mRNA data, the reduced mRNA level stimulated by 500 μM IS was significantly alleviated by RSV (Figure 4E). These data show that the phosphorylation of ERK1/2 and p38 MAPK has an inverse relationship with the activation of the IS/AhR pathway.

### 2.5. Role of ERK1/2 and p38 MAPK Signaling in Osteogenesis

**Figure 5:**
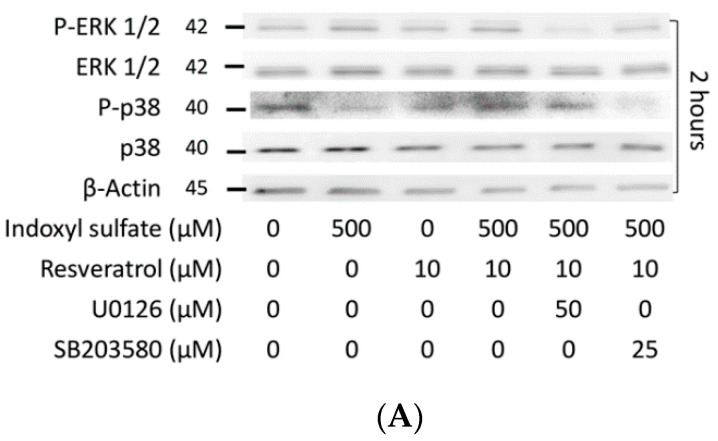

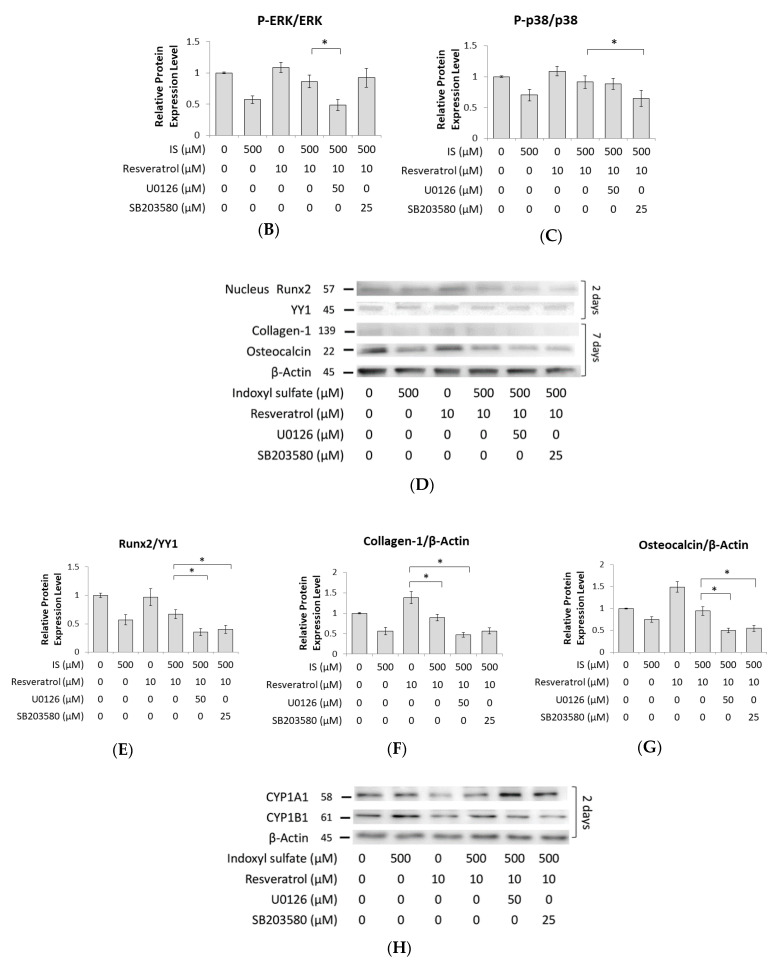

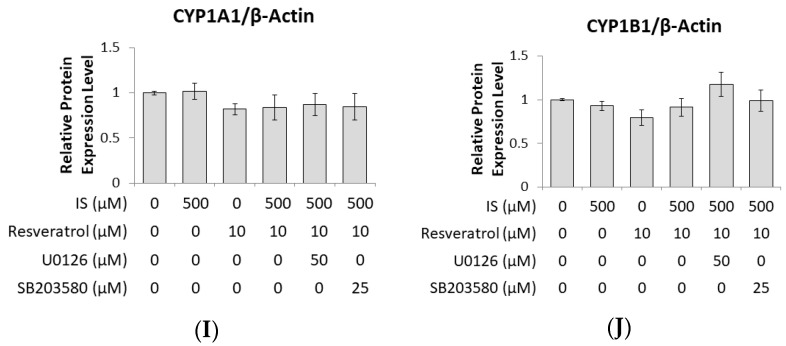
The effect of MAPK inhibitors on osteogenesis markers and the AhR pathway. After 1 h of U0126 or SB203580 stimulation, MC3T3 E1 cells were cultured in an osteogenesis medium with IS at 0, 100, 500, and 1000 μM with or without RSV. (**A**–**C**) After 2 h of culture at 500 μM IS and 10 μM RSV, ERK1/2 and p38 MAPK phosphorylation was significantly decreased in U0126 and SB203580, respectively. (**D**–**G**) After 2 or 7 days of culture, both U0126 and SB203580 significantly suppressed the protein expression of Runx2, collagen 1, and osteocalcin for IS and RSV at 500 and 10 μM, respectively. (**H**–**J**) After 2 days of culture, neither U0126 nor SB203580 influenced the protein expression of CYP1A1 and CYP1B1 at 500 μM with 10 μM RSV. Data are represented as the mean ± SEM (*n* = 3). * Indicates differences that were considered significant between the indicated groups (*p* < 0.05).

The importance of MAPK in osteoblast development was further verified using pharmacological inhibitors [16]. As expected, after 1 h of U0126 (ERK inhibitor) or SB203580 (p38 inhibitor) stimulation followed by 2 h of IS and RSV treatment, U0126 and SB203590 were observed to block ERK1/2 and p38 MAPK phosphorylation, respectively (Figure 5A–C). Moreover, the expression of osteogenesis markers (Runx2, collagen 1, and osteocalcin) was attenuated by U0126 or SB203580 in the group with 2 or 7 days of IS and RSV stimulation (Figure 5D–G). These data demonstrate that the suppressed ERK1/2 and p38 MAPK signaling induced by IS exposure can reduce the expression of important osteogenesis markers. In other words, osteoblast development is regulated via the ERK and p38 MAPK pathways.

Intriguingly, the protein expression of CYP1A1 and CYP1B1 in the groups with IS and RSV stimulation with or without ERK1/2 and p38 MAPK inhibitors remained the same (Figure 5H–J). This finding indicates that the inhibitors of ERK1/2 and p38 MAPK do not influence the expression of AhR.

### 2.6. AhR Knockdown Rescues the Downstream Expression of ERK, p38 MAPK, and Runx2

**Figure 6:**
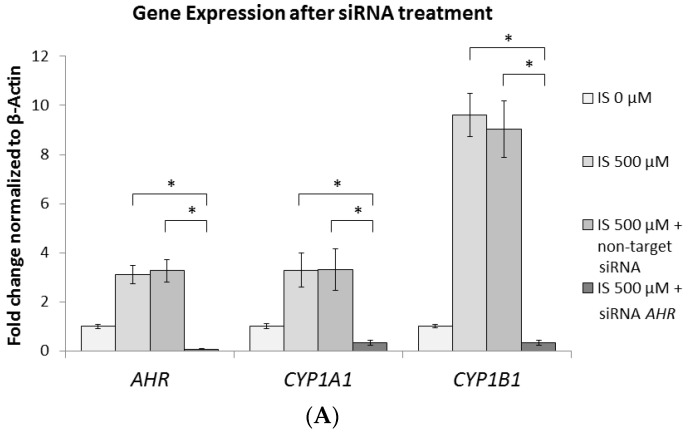

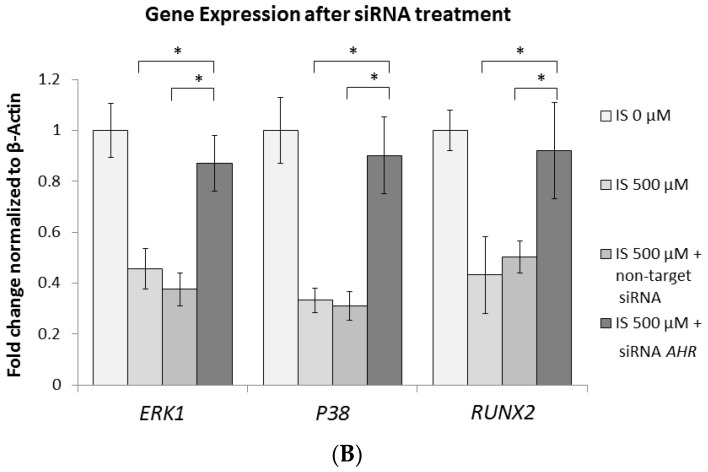
AHR Knockdown results in suppressed CYP1A1 and CYP1B1 gene expression and elevated ERK1, P38, and RUNX2 gene expression. After 1 day of pretreatment with small interfering RNA (siRNA) AHR, or non-target siRNA, MC3T3-E1 cells were cultured in an osteogenesis medium with IS at 0 or 500 μM for 2 days. (**A**) At 500 μM IS, the expression of AHR, CYP1A1, and CYP1B1 genes was significantly suppressed in the siRNA AHR group compared with the 500 μM IS alone and non-target groups. (**B**) At 500 μM IS, the gene expression of ERK1, P38, and RUNX2 was significantly enhanced in the siRNA AHR group when compared to the 500 μM IS alone and non-target groups. Data are represented as the mean ± SEM (*n* = 3). * Indicates differences that were considered significant between the indicated groups (*p* < 0.05).

Figure 3A–D and Figure 4A–C indicate an inverse correlation between the ERK1/2 and p38 MAPK pathways and AhR signaling under IS exposure. However, Figure 5H–J show that the expression of AhR was unaffected by ERK1/2 and p38 MAPK inhibitors. For further clarification of this relationship, we transfected MC3T3-E1 cells with small interfering RNA (siRNA) AHR and observed diminished mRNA expression of AHR, CYP1A1, and CYP1B1 (Figure 6A). By contrast, siRNA AHR enhanced the mRNA levels of not only ERK1 and P38 but also RUNX2 (Figure 6B). This indicates that AhR signaling occurs upstream of the ERK1/2 and p38 MAPK pathways. Therefore, the IS/AhR/ERK and p38 MAPK/Runx2 axis is a crucial signaling pathway for osteoblast differentiation in the context of CKD.

**Figure 7:**
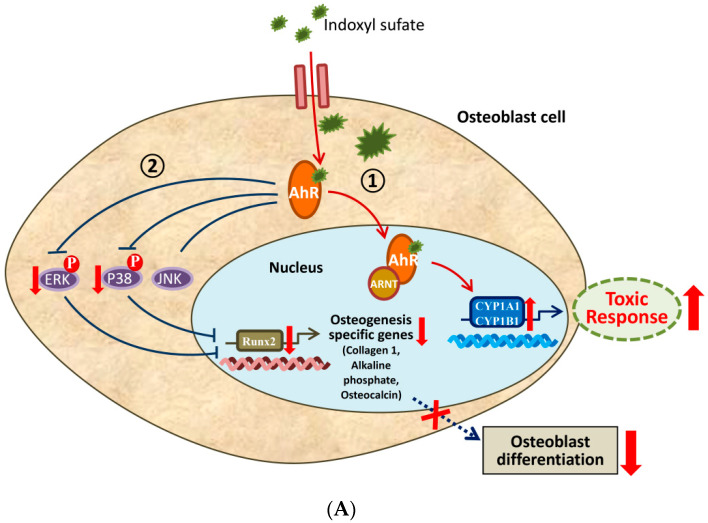

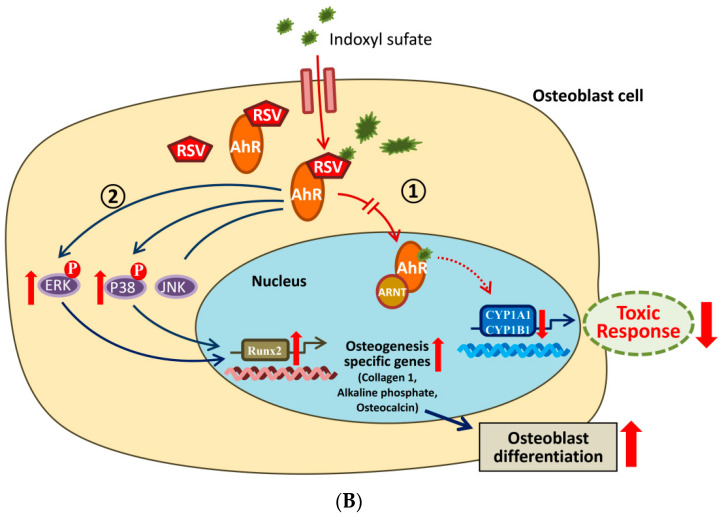
AhR downstream pathway in the cytoplasm. (**A**) ① IS can activate the classic function of AhR, increasing the expression of target proteins such as CYP1A1 and CYP1B1. ② IS can inhibit the phosphorylation of ERK and p38 MAPK through the AhR pathway in the cytoplasm. The binding of IS to AhR initiates a cytoplasmic signaling pathway that inhibits the phosphorylation of ERK and p38 MAPK, which then reduces the expression of Runx2 to impede osteoblast differentiation. (**B**) ① RSV, an AhR antagonist, blocks the activation of AhR and then diminishes the expression of CYP1A1 and CYP1B1. ② RSV can restore the reduction in ERK and p38 MAPK phosphorylation due to IS and then ameliorate the expression of Runx2 to promote osteoblast differentiation. P in red circle means phosphorylation of the substance. Upward red arrow indicates increase. Downward red arrow indicates decrease. Dotted blue or red arrow indicates weakened.

Overall, as described by the schemes shown in Figure 7, IS appears to attenuate osteoblast differentiation through the AhR/ERK1/2 and p38 MAPK/Runx2 pathways, and this effect is ameliorated by RSV.

## 3. Discussion

The term chronic kidney disease–mineral and bone disorder (CKD–MBD) is currently used to describe the conditions of inappropriate mineral and bone metabolism related to CKD [41]. The mechanisms of bone metabolism and osteoblast development in response to IS exposure have not yet been fully elucidated.

Our data emphasized that, in vivo, femur epiphysis reveals the disturbance of histological architecture and an irregular array in the chondrocyte near the epiphyseal plate in 5/6 nephrectomies of young adult mice under IS exposure. Moreover, RSV administration ameliorated the epiphyseal architecture and attenuated uneven alignment of the chondrocytes. Thus, we further studied the molecular mechanism of osteoblast differentiation and maturation to explore the possible RSV benefits of the signaling pathway on bony architecture and constitution. In vitro, we found that increasing IS led to the activation of AhR and a decrease in the MAPK/Runx2 pathway phosphorylation involved in the differentiation of osteoblasts in MC3T3-E1 cells.

In our previous research, we confirmed that IS induces impaired osteoclastogenesis via the AhR/NFATc1 pathway in a dose and time-dependent manner [4]. In addition, Fukagawa et al. found that high serum IS in CKD causes skeletal resistance to PTH, prohibits osteoblast cell cAMP production, and reduces the expression of the PTH receptor. As a result, CKD stimulates oxidative stress in osteoblasts [42]. However, Watanabe et al. demonstrated that IS aggravates low bone turnover disease in rats with parathyroidectomy and suggested that IS directly inhibits bone formation and is not relevant to skeletal PTH resistance [43]. In the present study, we found that the dysfunction of osteoblasts in CKD might be due to IS bone toxicity through the elevation of AhR expression and decreased phosphorylation in the ERK and p38 MAPK/Runx2 pathway of MC3T3-E1 cells.

Several researchers have discussed the key roles of AhR in bone development [23,44]. For example, TCDD activates AhR signaling and inhibits osteoblast differentiation from bone marrow-derived stem cells (BMSCs) [45], promotes deterioration of the bone remodeling process, and weakens bone strength [46]. In an in vivo study, mice treated with RSV presented an improvement with increased bone mineral density and bone mass [47]. In our study, we obtained similar results showing that AhR is influenced by IS and RSV. We demonstrated that 500 μM IS can enhance the expression of *AHR* mRNA, which could be attenuated by 10 μM RSV (Figure 3D). In addition, osteoblast development markers exhibited an opposite outcome when affected by IS and RSV (Figure 3G).

Conversely, Ryan et al. found that alkaline phosphatase expression was decreased in the AhR knockout mice [48], which suggests that AhR is indispensable for bone formation. These inconsistent research results can be explained by low doses of AhR with possible benefits for bone development, while the overexpression of AhR, in contrast, can impair bone formation. In our previous study examining osteoclast development, we found that low dose and short duration IS exposure can promote osteoclastogenesis through the AhR pathway. Conversely, high dose and long duration IS exposure can inhibit osteoclastogenesis through AhR by increasing the ubiquitination of NFATc1, which is a transcription factor in osteoclasts. These conflicting research results indicate that diverse AhR activators have opposite functions due to variations in their combining capacities, activated durations, or pathways resulting from divergent ligands.

Full knowledge of the MAPK pathways in osteoblasts is essential for realizing the physiologic control of bone formation. Of the three typical MAPKs, the activation of ERK and p38 was verified to have a predominant effect on bone development [17]. There are many in vivo and in vitro research studies on this factor [16,49]. In our study, we found that the phosphorylation of ERK and p38 MAPK, but not JNK, was suppressed by IS in osteoblast cells (Figure 4A–D).

Several studies have detected cross-talk between AhR and multiple transduction signaling, particularly the MAPK pathway [50]. TCDD and benzo[a]pyrene are conventional AhR agonists, which can also initiate the MAPK pathway [51]. Dou et al. reported that indole-3 acetic acid (IAA), another indolic uremic toxin, initiates the phosphorylation of ERK and p38 MAPK to increase the expression of tissue factor (TF) in human endothelial cells. Later, the authors demonstrated that IAA binds with AhR to induce the cytoplasmic p38 MAPK signaling pathway [52], which resulted in TF upregulation by IAA [53]. Furthermore, the authors demonstrated that p38 MAPK is downstream of AhR and also argued that this non-genomic AhR pathway is not related to ERK signaling [53]. In another study, Yu et al. demonstrated that TCDD activated AhR, which inhibited osteoblast proliferation and differentiation due to the Ahr-dependent activation of the ERK MAPK signaling pathway [54]. However, we found that the phosphorylation of ERK and p38 MAPK is then suppressed by IS, which disagrees with the above results acquired with IAA and TCDD. Moreover, RSV was able to alleviate the suppression of the phosphorylation of ERK and p38 MAPK under IS exposure in our study. 

Some evidence from previous reports has shown that RSV efficiently limits the induction of CYP1A1 via the AhR ligand [55]. In our data, we obtained a similar result, showing that RSV treatment can block the elevation of CYP1A1 and CYP1B1 in protein and mRNA levels induced by IS (Figure 3A–D). Dai et al. suggested that RSV (10^−8^~10^−5^ M) enhances the proliferation of human stem cells and the differentiation of osteoblastic cells in a time- and dose-dependent manner through ERK1/2 activation, which increases alkaline phosphatase activity and calcium deposition in human BMSC cultures [14]. In our study, we also found that the suppression of osteoblast development markers by IS could be restored by RSV (Figure 3G).

In a study of Tan et al., the authors demonstrated that both the ERK and JNK MAPK pathways contribute to TCDD-induced AhR activity by applying the inhibitors of MAPK activity to interfere with the the AhR-dependent gene [56]. In our study, through the inhibitors of ERK1/2 and p38 (U0126 and SB203580), we found that the phosphorylation of both ERK1/2 and p38 was suppressed (Figure 5A–C), but CYP1A1 and CYP1B1 were not reduced (Figure 5H–J). To verify that AhR is located upstream of ERK1/2 and p38 MAPK, we administered siRNA *AHR* to MC3T3-E1 cells under IS exposure; the suppressed gene expression of *ERK1* and *P38* was also restored. This demonstrates that AhR signaling occurs upstream of the MAPK pathway.

In conclusion, our current study is the first to demonstrate that the IS/AhR/MAPK/Runx2 signaling pathway in MC3T3-E1 cells disturbs osteoblast differentiation and that RSV blocks this pathway to restore osteogenic development. The results of the present study thus provide the basis for a novel treatment strategy involving the enhancement of bone quality in CKD-MBD using RSV. However, further studies are needed to understand the complex signaling pathways related to RSV.

## 4. Materials and Methods

### 4.1. Reagents and Chemicals

All culture media components, including α-MEM, fetal bovine serum, and antibiotic–antimycotic (100×), were acquired from Gibco (Las Vegas, NV, USA). An osteoblast differentiation medium containing L-ascorbic acid, β-glycerophosphatase, and dexamethasone was obtained from Sigma (St. Louis, MO, USA). The indoxyl sulfate (IS) and resveratrol (RSV) used in the osteoblast treatment were acquired from Sigma (St. Louis, MO, USA). The primary antibodies were obtained as indicated: anti-AhR (Santa Cruz, Dallas, TX, USA), anti-CYP1A1 (GeneTex, Hsinchu City, Taiwan), anti-CYP1B1 (GeneTex, Hsinchu City, Taiwan), anti-MAPK series (ERK1/2, p38, JNK, phospho-ERK1/2, phospho-p38, and phospho-JNK) (Cell Signaling, MA, USA), anti-Runx2 (Abcam, Cambridge, UK), anti-collagen 1 (GeneTex, Hsinchu City, Taiwan), anti-osteocalcin (Santa Cruz, Dallas, TX, USA), and anti-β Actin (Proteintech, Rosemont, IL, USA). Horse Radish peroxidase (HRP)-conjugated anti-rabbit immunoglobulin G and anti-mouse immunoglobulin G were obtained from the Proteintech Group (Chicago, IL, USA). The qPCR reagents included the RNA Extractor kit (Tools, Taiwan), DNase I Amplification Kit (Invitrogen, USA), iScript cDNA Synthesis Kit (Bio-Rad, Hercules, CA, USA), and Simply Green qPCR Master Mix (GeneDireX Inc., Taichung City, Taiwan).

### 4.2. In Vivo Study

All animal studies were approved by the Institutional Animal Care and Use Committee (IACUC-18-041, approved on 12 February 2018) and conducted in accordance with the National Institutes of Health Guidelines. BALB/c male mice (6 weeks old with a body weight of more than 20 g) with 5/6 nephrectomy (Nx) or sham surgery were obtained from BioLASCO Taiwan Co., Ltd (Taipei, Taiwan) and split into three groups (sham + normal saline (N/S) injection (*n* = 6), 5/6 Nx + N/S injection (*n* = 6), and 5/6 nephrectomy + resveratrol (RSV) injection (*n* = 6)) to establish chronic kidney disease models (IACUC-18-041). The 5/6 nephrectomy surgery was applied when the mice were 4 weeks old to remove 2/3 of the right-side kidney; one week later, the whole left side of the kidney was removed. At the same time, those in the sham group only underwent a back incision on both sides. All mice were kept in pathogen-free animal facilities under controlled conditions of 22 °C with a 12 h light/dark cycle. The mice were allowed to acclimatize for the first 4 weeks to establish the CKD model. When the mice were 10 weeks old and the serum creatinine and BUN levels of the 5/6 nephrectomy mice were higher than normal (Figure 1A,B), the mice received an intraperitoneal (IP) injection with N/S (110 λ/mouse) or an RSV (30 mg/kg/day, about 110 λ/mouse) treatment every day for 4 weeks. Then, the mice were sacrificed, and their bones were analyzed.

#### 4.2.1. Serum Creatinine (Scr) and Blood Urea Nitrogen (BUN) Measurement

Blood samples were collected from a tail vein and transferred into heparinized micro-tubes. The serum was isolated by centrifugation for 20 min at 1000 *g* in 4 °C; then, analysis occurred over 2 h. The levels of Scr and BUN were measured by the blood chemistry tests of the Taiwan Mouse Clinical (National Phenotyping Center, Taipei, Taiwan).

#### 4.2.2. H&E and IHC Staining

Briefly, distal femurs were fixed with 4% paraformaldehyde (Bio Basic, Toronto, Canada) and decalcified in 15% EDTA for a week. After dehydration through a series of ethanol solutions and a wax embedding procedure, the bones were sawn with 5 μm sections parallel to the long axis. For Hematoxylin and Eosin staining (H&E staining), after deparaffinization and rehydration, the sliced tissue was stained for 5 min with hematoxylin solution followed by 3 min with eosin solution (ScyTek Laboratories; Logan, UT, USA). For immunohistochemical (IHC) staining, the deparaffinized sliced tissue was incubated in a 60 °C water bath for 24 h to retrieve the antigen. Then, the tissue was covered with the Runx2 primary antibody (1:50) at 4 °C overnight. The following day, Rabbit Probe HRP Chromogen with DAB Brown (horse radish peroxidase developed with 3,3’-Diaminobenzidine) (BioTnA Biotech, Kaohsiung, Taiwan) was applied to the tissue for 30 min, followed by 3 min of hematoxylin staining. Stained images were observed under a microscope (Carl Zeiss, Germany) with different random fields in each group, and the Axio Vision Measurement Program by Axio cam MRc (Carl Zeiss, Germany) was used to analyze the brown regions.

#### 4.2.3. Micro-CT Analysis

For the Micro-CT analysis, femurs were fixed with 4% paraformaldehyde and scanned by quantitative micro-CT (Quantum FX µCT, Hopkinton, MA, USA) at a FOV 10 mm resolution and a pixel size of 20.0 µM. Various angles of isotropic bone voxels were analyzed with the NRecon Reconstruction Software (Allentown, PA, USA), and the data are presented according to four characteristics: bone volume per total volume (BV/TV), mean trabecular number (Tb. N.), mean trabecular thickness (Tb. Th.), and mean trabecular separation (Tb. Sp.).

### 4.3. In Vitro Study

To address the objectives outlined above, two kinds of cells were used: primary mouse osteoblastic cells and MC3T3-E1. Both kinds of osteoblastic cells were maintained in α-MEM, 10% fetal bovine serum, 1% antibiotic–antimycotic (100×), 100 μg/mL L-ascorbic acid phosphatase, 10 mM β-glycerophosphate, and 100 nM dexamethasone at 37 °C in a 5% CO2 humidified atmosphere.

#### 4.3.1. Primary Culture of Osteoblastic Cells

Primary osteoblastic cells were prepared from the calvariae of a neonatal mouse (IACUC-18-041). One-day-old BALB/c mice delivered by timed pregnant mice (BioLASCO, Taipei, Taiwan) were sacrificed, and their calvariae were dissected without suture tissues in a sterile manner. The dissected calvariae were rinsed in phosphatase-buffered saline (PBS) from GE Healthcare Life Science (Marlborough, MA, USA) and soaked with 1 mL of 0.05% trypsin from Corning Life Sciences (Union City, CA, USA)/0.1 mM EDTA from Omics Bio (Taichung City, Taiwan) containing 5 mg collagenase/Collagenase P from Roche Applied Science (Indianapolis, IN, USA) 10 times for 10 min each. Cells were then collected from the collagenase medium. Osteoblastic cells were recognized by alkaline phosphatase staining (Cosmo Bio; Tokyo, Japan) at 14 days (Figure 2B).

#### 4.3.2. MC3T3-E1 Cell Line

The MC3T3-E1 Subclone 14 cell line was obtained from American Type Culture Collection (Manassas, VA, USA).

#### 4.3.3. Cell Viability

Primary osteoblast cells were seeded in 96-well plates (1 × 10^3^ cells/well) with an osteogenic induction medium for 24 and 48 h and were treated at different concentrations (0, 20, 100, 250, 500, and 1000 μM) of IS with or without 10 μM RSV. The working RSV concentration was set according to an osteoblast pilot study [57]. The cell viability was measured using a Cell Counting Kit-8 (CCK-8) assay kit (Dojindo Molecular Technologies, Inc.; Rockville, MD, USA), as previously described [4].

#### 4.3.4. Alkaline Phosphatase Staining and Alkaline Phosphatase Activity

Primary osteoblast cells were seeded in 24-well plates (3 × 10^3^ cells/well) or 6-well (1 × 10^5^ cells/well) plates with different concentrations (0, 20, 100, 250, 500, and 1000 μM) of IS, with or without 10 μM RES, for observation on day 14. The cells of the 24-well plates were fixed with 4% paraformaldehyde for 15 min and incubated with an alkaline phosphatase staining kit from Cosmo Bio (Tokyo, Japan). To check the activity of alkaline phosphatase, the cells of the 6-well plates were assayed using an alkaline phosphatase assay kit (Colorimetric) from Abcam (Cambridge, UK). As per the manufacturer’s instructions, the cells were appropriately lysed from each well to obtain the cell lysate containing equal cell protein amounts. The cell lysate with para-nitrophenyl phosphatase (pNPP) was incubated in a 96-well plate at 25 ˚C protected from light. After 60 min, the enzyme converted the pNPP substrate into an equal amount of colored p-nitrophenol (pNP), which was measured at O.D. 405 nm. The alkaline phosphatase activity in the test samples was calculated as the amount of pNP in a sample well calculated from a standard curve (µ mol)/reaction time (mins)/10^5^ cells.

#### 4.3.5. Alizarin Red Staining and Quantification Assay

Primary osteoblast cells were seeded in 24-well plates (3 × 10^3^ cells/well) for 21 days. The Alizarin Red S Staining Quantification Assay (Ared-Q) was purchased from Sciencell Research Laboratories (Carlsbad, CA, USA). The cells were fixed with 4% paraformaldehyde for 15 min and incubated with Alizarin Red S (ARS) reagent at room temperature for 30 min. For the analysis of quantification, 10% acetic acid was added to each well for 30 min with shaking at room temperature. Collected samples were transferred to micro-centrifuge tubes with heating at 85 °C for 10 min and then placed on ice for 5 min. After centrifuging at 20,000 g for 15 min, samples were neutralized with 10% ammonium hydroxide, and the absorbance of an aliquot was read at 405 nM using a plate reader.

#### 4.3.6. Western Blot Analysis

Protein from MC3T3-E1 whole cells was extracted with a RIPA buffer from Bio Basic Inc. (Toronto, Canada) containing a broad-spectrum protease inhibitor cocktail from BIONOVAS (Toronto, Canada). Moreover, a Nuclear/Cytosol Fractionation Kit from BioVision, Inc. (Milpitas, CA, USA) was used to separate the nucleus protein and the cytoplasmic protein from the whole cells. As described in previous research [4], the protein samples were each loaded into a well of an SDS–PAGE gel followed by electrophoresis and then transferred to a polyvinylidene fluoride membrane. The membrane was incubated in primary antibodies overnight at 4 °C, followed by secondary antibody incubation for 1 h at room temperature. Images were taken with an ECL substrate from GE Healthcare (Chicago, IL, USA). The relative intensity level of each protein band was quantified by the ImageJ software (NIH, Bethesda, MD, USA) [58].

#### 4.3.7. qPCR and si-RNA AhR

Total cellular RNA was extracted from the cells using an RNA Extractor kit from Tools (New Taipei City, Taiwan) and quantified using a Nanodrop 2000 device (Thermo Fisher; Waltham, MA, USA), as previously described [4]. cDNA was reverse transcribed using an iScript cDNA Synthesis Kit. The real-time quantitative polymerase chain reaction (qPCR) was conducted using the LightCycler® 480 Instrument II (Roche Molecular Systems, Inc.; Pleasanton, CA, USA) to measure the expression levels of the genes of the osteoblast development markers. The qPCR primers are listed in Table 1 of the Supplementary Material. All primers were synthesized by Genomics (Taipei, Taiwan). The mRNA expression level of β-actin was used as an internal control to normalize the gene expression in each sample.

To evaluate the effects of AHR transcription, small interfering RNA (siRNA) oligonucleotides directed against mouse *AHR* (si-RNA AHR; siGENOME Mouse *AHR* siRNA), siGENOME Non-Targeting siRNA, and DharmaFECT Transfection Reagent 1 were obtained from Dharmacon (Horizon Discovery Group Company, Waterbeach, UK). There were four groups of MC3T3-E1 cells cultured in 24-well plates under different simulative conditions of 0, 500, and 500 μM IS with Non-Targeting siRNA and 500 μM IS with siRNA *AHR*. The siRNA transfections were performed in each well with siRNA *AHR* (5 nmol) or Non-Targeting siRNA (5 nmol) added into 2.0 μL of a TransFectin reagent with α-MEM to a final volume of 500 μL. After siRNA transfection for 24 h, the transfected cells were cultured in an antibiotic-free osteogenic induction medium for 48 h. Then, changes in the levels of mRNA between groups were checked.

### 4.4. Statistical Analysis

Representative data are presented as the mean ± standard deviation (SD), and at least three independent experiments were conducted per condition. The data were analyzed using the SAS 9.0 software (SAS Institute Inc., Cary, NC, USA), and *p* < 0.05 was considered to be significant.

## Figures and Tables

**Table 1:** List of primer sequences used for qRT-PCR analysis in this study.

Gene.	Forward Primer 5′→3′	Reverse Primer 5′→3′
*AHR*	TTCTTAGGCTCAGCGTCAGCTA	GCAAATCCTGCCAGTCTCTGAT′
*Alkaline Phosphatase*	GAGCGTCATCCCAGTGGAG	TAGCGGTTACTGTAGACACCC
*Collagen 1*	GAGCGGAGAGTACTGGATCG	GCTTCTTTTCCTTGGGGTTC
*CYP1A1*	GTCAGGACAGGAAGCTGGAC	GAGGCTCCACGAGATAGCAG
*CYP1B1*	GCCACTATTACGGACATCTTCGG	ACAACCTGGTCCAACTCAGCCT
*ERK1*	GACACCCCTGTCCTTTTGGATCTGGTCCTG	AAGCAGAGACCCCAGCAAAGTGAGAGAAG
*Osteopontin*	CCGCTCGAGACCATGAGATTGGCAGTGAT	CGGGATCCCTAGTTGACCTCAGAAGATG
*p38*	GAAAGTGTAAAGCCAATTCCAGTGTTGGAC	GTCTGCCTCCTTCTGGGCTCCAAATGATTC
*RUNX2*	TTCAACGATCTGAGATTTGTGGG	GGATGAGGAATGCGCCCTA
*β-Actin*	CCTCTATGCCAACACGTGC	CCTGCTTGCTGATCCACATC

## Abbreviations

AhR	Aryl hydrocarbon Receptor
ARNT	Aromatic hydrocarbon Receptor Nuclear Transfer Protein
BMSCs	Bone Marrow-derived Stem Cells
BUN	Blood Urea Nitrogen
BV/TV	Bone Volume over Total Volume
CKD-MBD	Chronic Kidney Disease – Mineral and Bone Disorder
CYP1A1	Cytochrome P450 family 1 Subfamily A Member 1
CYP1B1	Cytochrome P450 family 1 Subfamily B Member 1
DAB	3,3’-Diaminobenzidine
H&E staining	Hematoxylin and Eosin Staining
IHC staining	Immunohistochemical staining
IS	Indoxyl Sulfate
HRP	Horse Radish peroxidase
MAPK	Mitogen-activated Protein Kinase
N/S	Normal Saline
Nx	Nephrectomy
PTH	Parathyroid Hormone
RSV	Resveratrol
Runx2	Runt-related Transcription Factor 2
siRNA	Small Interfering RNA
Tb.N.	Trabecular Number
Tb.Sp.	Trabecular Separation
Tb.Th.	Trabecular Thickness
TCDD	2,3,7,8-tetrachlorodibenzo-p-dioxin
